# Metabolite and transcript profiling of berry skin during fruit development elucidates differential regulation between Cabernet Sauvignon and Shiraz cultivars at branching points in the polyphenol pathway

**DOI:** 10.1186/s12870-014-0188-4

**Published:** 2014-07-26

**Authors:** Asfaw Degu, Uri Hochberg, Noga Sikron, Luca Venturini, Genny Buson, Ryan Ghan, Inbar Plaschkes, Albert Batushansky, Vered Chalifa-Caspi, Fulvio Mattivi, Massimo Delledonne, Mario Pezzotti, Shimon Rachmilevitch, Grant R Cramer, Aaron Fait

**Affiliations:** 1 The Albert Katz International School, Beer-Sheva, Israel; 2 The French Associates Institute for Agriculture and Biotechnology of Drylands, the Jacob Blaustein Institute for Desert Research, Ben-Gurion University of the Negev, Sede Boqer 84990, Israel; 3 Biotechnology Department, University of Verona, Strada Le Grazie 15, Verona, Italy; 4 Department of Biochemistry and Molecular Biology, University of Nevada, Reno 9557, NV, USA; 5 The National Institute for Biotechnology in the Negev, Ben-Gurion University of the Negev, Beer-Sheva, Israel; 6 Department of Food Quality and Nutrition, Research and Innovation Centre, Fondazione Edmund Mach, San Michele all’Adige, Italy

**Keywords:** Metabolite profiling, Grape berry metabolism, Grapevine, Transcript analysis, Metabolomics, GC-MS, LC-MS

## Abstract

**Background:**

Grapevine berries undergo complex biochemical changes during fruit maturation, many of which are dependent upon the variety and its environment. In order to elucidate the varietal dependent developmental regulation of primary and specialized metabolism, berry skins of Cabernet Sauvignon and Shiraz were subjected to gas chromatography–mass spectrometry (GC-MS) and liquid chromatography–mass spectrometry (LC-MS) based metabolite profiling from pre-veraison to harvest. The generated dataset was augmented with transcript profiling using RNAseq.

**Results:**

The analysis of the metabolite data revealed similar developmental patterns of change in primary metabolites between the two cultivars. Nevertheless, towards maturity the extent of change in the major organic acid and sugars (i.e. sucrose, trehalose, malate) and precursors of aromatic and phenolic compounds such as quinate and shikimate was greater in Shiraz compared to Cabernet Sauvignon. In contrast, distinct directional projections on the PCA plot of the two cultivars samples towards maturation when using the specialized metabolite profiles were apparent, suggesting a cultivar-dependent regulation of the specialized metabolism. Generally, Shiraz displayed greater upregulation of the entire polyphenol pathway and specifically higher accumulation of piceid and coumaroyl anthocyanin forms than Cabernet Sauvignon from veraison onwards. Transcript profiling revealed coordinated increased transcript abundance for genes encoding enzymes of committing steps in the phenylpropanoid pathway. The anthocyanin metabolite profile showed F3′5′H-mediated delphinidin-type anthocyanin enrichment in both varieties towards maturation, consistent with the transcript data, indicating that the F3′5′H-governed branching step dominates the anthocyanin profile at late berry development. Correlation analysis confirmed the tightly coordinated metabolic changes during development, and suggested a source-sink relation between the central and specialized metabolism, stronger in Shiraz than Cabernet Sauvignon. RNAseq analysis also revealed that the two cultivars exhibited distinct pattern of changes in genes related to abscisic acid (ABA) biosynthesis enzymes.

**Conclusions:**

Compared with CS, Shiraz showed higher number of significant correlations between metabolites, which together with the relatively higher expression of flavonoid genes supports the evidence of increased accumulation of coumaroyl anthocyanins in that cultivar. Enhanced stress related metabolism, e.g. trehalose, stilbene and ABA in Shiraz berry-skin are consistent with its relatively higher susceptibility to environmental cues.

## Background

Grape cultivars evolved from many origins [1] and were distributed to geographical regions with different climates [2] leading to the evolution of varietal specific physiology, morphology, growth and development [3],[4]. Grapevine vegetative growth and canopy structure affects the amount of light reaching the grape bunch and modifies its microclimate [5]-[7]. Moreover, the partitioning of photoassimilates and other metabolites to the bunch will also depend on the extent of vegetative growth during berry development and eventually it will determine the metabolite profile of the flesh and skin of the berry [5],[8],[9]. Hence, a complex interaction between genotype, macro- and micro-climate and plant vigor will define berry metabolism and eventually wine quality [10].

Grape berry, a non-climacteric fruit [11], follows a double sigmoidal growth pattern which is composed of three major phases [12], each characterized by changes in size, color, texture, metabolic composition and gene expression [13],[14]. The hard green stage, phase I, is characterized by rapid cell growth and elongation with a concomitant massive accumulation of organic acids, hydroxyl cinnamic acid, amino acids and tannins towards the end of this phase [15]-[19]. In phase two, berry enlargement ceases, sugars start to accumulate at the expense of acids eventually leading to a rise in pH [15]. During veraison, berries enter a second rapid growth phase with a progressive decline in acidity and increase in softness. Veraison is a stage that marks the beginning of phase III, it is associated with berry softening and fundamental metabolite changes such as the accumulation of sugars, aroma precursors and pigments, [15],[16] as well as other classes of specialized metabolites that will contribute to berry quality [20]. The rate of biochemical changes during berry development is varietal dependent [21]-[23] as well as influenced by climatic factors [24]-[26], soil [27], irrigation [28]-[31] and viticultural practices [32]-[34].

The role of secondary metabolism in berries is not understood completely. Plants with fleshy and tasty fruits were likely favored during evolution by animal driven dispersal [35] and the pericarp and skin possibly evolved as protective tissues for developing seed [36]. As such, polyphenols, including numerous flavonoids as well as non-flavonoid compounds, play a central protective role in plant adaptation to the environment [37]. Flavonols protect berries from UV radiation [38], anthocyanins play a role in seed dispersal, provide protection from high temperature and UV radiation, and proanthocyanidins posses antifungal properties [39]-[41]. Flavonoids are biologically active compounds due to their antioxidant activities [42],[43] and contribute to organoleptic properties of the fruit and wine [16],[44].

Since the draft sequence of the Pinot Noir grapevine genome [45], the regulation of berry development and the associated metabolic processes were the focus of an increasing number of whole-genome gene expression studies [14],[15],[46]-[50]. Nevertheless, there are extensive gaps in our understanding of the regulation of secondary metabolism and the differences in secondary metabolism amongst different cultivars. For example, recent transcriptomic and genomic studies on different commercial clones discovered hundreds of genes missing from the Pinot Noir reference genome [51]-[53]. Thus, more comparative studies are needed to fully elucidate the regulation of grape metabolism during berry development. Recently developed RNA-seq transcriptome profiling approaches, provides higher resolution and capability of detecting different isoforms of a transcript compared to the microarray based methods [54]. To date, there are only a few studies in grape berry development that were performed using RNAseq [55]-[57] and none in comparative studies. Moreover, given the significant post-transcriptional regulation of fruit metabolism [58]-[61], transcriptional studies are increasingly being supported by- and integrated with metabolic and proteomic data. Large-scale metabolite profiling has been applied to grape only recently [15],[21],[62]-[65] and mostly using targeted analysis of primary or secondary metabolism.

In the present study we used GC-MS and LC-MS based metabolic profiling of the berry skin to study the developmental processes characteristic of two red wine grape varieties Shiraz (SH) and Cabernet Sauvignon (CS) grown in the field in a semiarid environment in southern Israel. The gas chromatography–mass spectrometry (GC-MS) and liquid chromatography–mass spectrometry (LC-MS) generated dataset was augmented with RNAseq-transcript profiles. The study aimed to examine varietal differences in the metabolism of developing berries and the underlying regulatory mechanism.

To the best of our knowledge, this is the first attempt to integrate metabolomics and RNAseq-based transcriptome data in a comparative study of berry development on two physiologically contrasting cultivars in grape.

## Methods

### Plant material and experimental conditions

The field experiment was conducted on four-year old commercial vines of two red wine grape varieties of *Vitis vinifera* L. (Shiraz and Cabernet Sauvignon) grafted on Ruggeri during the 2011 growing season. Both varieties were planted with 3 m spacing between rows and 1.5 m between vines. The vines were trained onto vertically oriented trellis in a East–west orientation. The vineyard is located at 30.68 N 34.80E; with an average rainfall of 86 mm and an average potential evaporation of over 2000 mm. Soil water content was measured on an hourly basis and it is given in Additional file 1: Figure S1. Irrigation was applied every four days based on evaporation from evaporation pan Class A (ET_pan_) using an irrigation factor of 0.25. Plants were drip irrigated using in-line emitters at a flow rate of 2.2 L h^−1^. To avoid any edge effects, only plants located in the middle of each row were used for the experiment. The experimental plot was bordered with vine row receiving a similar irrigation amount.

### Berry quality related traits

Prior to each sampling, total soluble solids (TSS) in ^o^BRIX were assayed from the berry juice of five different vines using a hand refractometer (Palette PR-100, Atago, USA). Berry weight was averaged from at least 15 berries selected from the middle part of representative bunches from eight plants of each cultivar on both sides (N and S) of the row. Titratable acidity measurement (g/L tartaric acid equivalents) was performed on the same samples according to the standard procedures used in Guymon and Ough [66].

### Sampling and metabolite extraction

Samples were taken for metabolic analysis at four developmental stages in both cultivars (number of days are given in reference to veraison stage): 30 days before veraison (DTV) (pre-veraison); veraison; 20 days after veraison (DAV) (post-veraison) and harvest 37 DAV for Shiraz and 53 DAV for CS. Shiraz and CS were differentially sampled at harvest in relation to the ^o^BRIX measured. At all sampling dates, five berries representative of the middle portion of the bunch and at similar developmental stage were collected on both N and S side of a given vine-plant and pooled together. Six independent biological replicates, each consisting of the pooled berries from six separate vines, were collected in the middle section of the row in both cultivars. Skin was carefully peeled from the berries, gently removing the remaining flesh from the skin with a clean cloth. Immediately after, the tissue was snap-frozen in liquid nitrogen and kept at −80°C until further analysis. Ahead of extraction samples were freeze dried in a lyophilizer (VIRTIS GARDINER, N.Y. R525, Model 10-MR-TR). The freeze-dried samples were extracted for parallel metabolite profiling (LC and GC/MS) using a slightly modified version of the method described previously [67]. All chemicals were purchased from Sigma-Aldrich if not indicated otherwise. Berry skin tissue was ground using a RETCH-mill (Retsch Gmbh, 42787 Haan, Germany) with pre-chilled steel holders and grinding beads. For metabolite extraction, 70 mg of frozen powder were weighed and extracted in a pre-chilled methanol: chloroform: water extraction solution (2.5:1:1 v/v). Internal standards, (i.e. 0.2 mg/ml ribitol in water, 1 mg/ml ampicillin in water and 1 mg/ml corticosterone in methanol), were subsequently added. The mixture was then briefly vortexed, centrifuged for 2 min at 14000 RPM (microcentrifuge 5417R) and the supernatant was decanted into the new tubes. The supernatant was mixed with 300 μl of chloroform (LC/MS grade) and 300 μl of UPLC-grade water and then centrifuged at 14,000 RPM for 2 min. After that, 100 μl of the water/methanol phase was dried in a vacuum concentrator (Eppendorf Concentrator Plus) for derivatization [68] for GC-MS analysis. The remaining water/methanol phase was transferred to UPLC vials for LC-MS analysis.

### GC-MS derivatization and data processing

GC-MS samples from the above extraction were re-dissolved and derivatized. Eight microliters of a retention time standard mixture (0.029% v/v *n*-dodecane, *n*-pentadecane, *n*-nonadecane, *n*-docosane, *n*-octacosane, *n*-dotracontane, and *n*-hexatriacontane dissolved in pyridine) was added. The sample set also included a reference quality control of authentic metabolite standards (1 mg ml^−1^ each) (Additional file 2: Table S1A). Volumes of 1 μL were then injected onto 30-m VF-5 ms GC column with 0.25 mm i.d., film thickness of 0.25 μ m, and + 10 m EZ-Guard (Agilent) in splitless and split mode (32:1) allowing a more accurate comparison of highly abundant metabolites (e.g. tartarate, sugars, and inositol). The GC-MS system consisted of an AS 3000 autosampler, a TRACE GC ULTRA gas chromatograph, and a DSQII quadrupole mass spectrometer (Thermo-Fisher ltd). The parameters of the machine were exactly as described in [69]. Spectral searching was done by consulting the National Institute of Standards and Technology (NIST, Gaithersburg, USA) algorithm incorporated in the Xcalibur® data software (version 2.0.7) against RI libraries from the Max-Planck Institute for Plant Physiology in Golm, Germany (http://www.mpimp-golm.mpg.de/mms-library/) and finally normalized by the total metabolites and correcte d for the dilution factor.

### UPLC–MS analysis

For LC-MS analysis, 4 μl of extracted sample was injected onto a UPLC-QTOF-MS system equipped with an ESI interface (Waters Q-TOF Xevo^TM^: Waters MS Technologies, Manchester, UK) operating in negative and positive ion modes. Chromatographic separation was carried out on an Acquity UPLC BEH C_18_ column (100 mm × 2.1 mm, 1.7 μm). The column and autosampler were maintained at 40°C and 10°C, respectively. During each sample running, the mobile phase comprised 95% water, 5% acetonitrile, 0.1% formic acid (phase A), and 0.1% formic acid in acetonitrile (phase B). The solvent gradient program was conditioned exactly as described previously [69]. All analyses were acquired using leucine enkephalin for lock mass calibration to ensure accuracy and reproducibility, at a concentration of 0.4 ng L^−1^, in 50/50 of acetonitrile/H_2_O with 0.1% v/v formic acid. The MS conditions were set essentially as described previously [69].

### UPLC data processing

MassLynxTM software (Waters) version 4.1 was used as the system controlling the UPLC and for data acquisition as described previously [69]. The raw data acquired were processed using MarkerLynx application manager (Waters) essentially as described previously [69]. To verify metabolite identification, representative samples of different developmental stages from each cultivar were run using the same instruments and under the same operating conditions at the metabolomics facility of the Edmund Mach Foundation in San Michele all’Adige – Italy, where an in-house standard library described in details in [70] was used to validate the annotation of the identified metabolites based on retention time order of commercial standards (Additional file 2: Table S1B), and metabolites were also identified based on a fragmentation pattern searched against the Chemspider metabolite database (http://www.chemspider.com/) and further confirmed with previous metabolite annotations [23],[71]-[76].

### Statistical analysis

The normalized data set (to tissue dry weight and internal standards) was subjected to Kruskal-Wallis and Wilcoxon rank sum tests to identify metabolites changing significantly during berry development. Comparisons between cultivars at each sampling date were performed using the Student’s *t*-test. Pairwise correlations to all annotated metabolites were calculated using the Pearson correlation algorithm. The corresponding *p*-values were computed using the cor.test function of R as previously described [77]. To avoid false positives, false discovery rate (FDR) at a Q value of 0.05 was performed. The correlation matrices were used as a data for network construction and analysis using “igraph” package (http://igraph.org/r/#docs). All the statistical analyses and network properties were calculated using the R-software environment R 3.0.1 (http://cran.r-project.org/).

### RNA-seq analysis

To facilitate parallel comparisons to the metabolite data, frozen, ground skin tissues from the same samples used for metabolite extraction were used. Total RNA was extracted from three biological replicates of 70 mg of berry skin tissue at veraison and 20 DAV in the two cultivars essentially as described by Japelaghi et al. [78]. The quality and concentration of extracted RNA was determined using Bioanalyzer Chip RNA 7500 series II (Agilent, Santa Clara, CA) and a Nanodrop 2000 spectrophotometer (Thermo Scientific, Wilmington, DE). Following quality assessment, poly (A) mRNA preparation and sequencing with an Illumina HiSeq 1000 sequencer (Illumina Inc., San Diego, CA, USA) were performed as described previously [52]. The resultant reads were aligned to the reference *Vitis vinifera* genome using TopHat software (version 2.0.6), which received as input the *Vitis vinifera* GTF file (option “-G”) and also using the following parameters: “–b2-very-sensitive –r 150 –mate-std-dev 50” [79]. Subsequently, the Cufflinks software [80]; version 2.0.2) was used to assemble aligned RNA-seq reads into transcripts with the parameters “--min-intron-length 10”; these assemblies were processed into a full transcriptome set by CuffMerge [81]. Finally, their abundance was estimated with CuffDiff [80]. In order to facilitate easy comparison and visualization of transcriptomic changes between developmental stages and the two cultivars, the data were normalized to the CS veraison stage transcript level.

### Functional categorization

Functional enrichment analysis of differentially expressed transcripts of CS and Shiraz was carried out based on VitisNet molecular networks [82]). The complete set of networks and their corresponding genes was downloaded from http://www.sdstate.edu/ps/research/vitis/upload/AdditionalFile2_2.xlsx. Enrichment against these networks was performed using a hyper-geometric test in Expander [83], Enrichment *p*-values were adjusted for multiple testing using the Bonferroni correction.

## Results

### Sampling stages and berry quality traits

**Figure 1:**
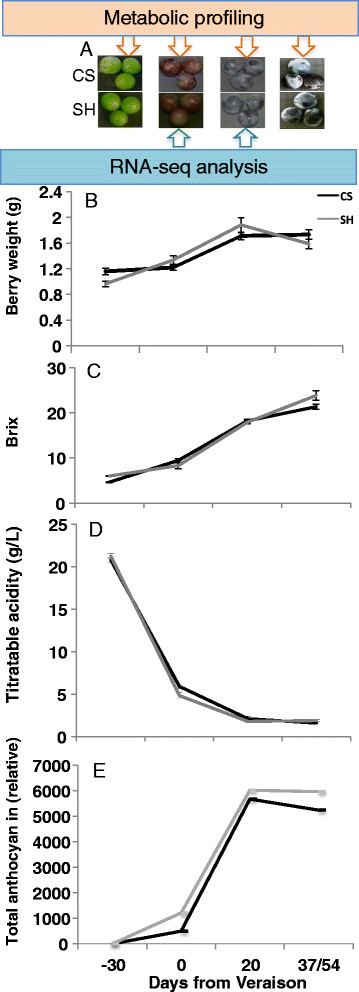
Sampling stages (A), berry weight measurements (B), BRIX (C), titratable acidity (D), and total anthocyanin (E) of the two cultivar at four berry developmental stages.

Berries sampled from the two cultivars had a similar pattern of weight increment during development and expected changes of other berry quality related parameters, including TSS and ^o^BRIX (Figure 1). Nevertheless, close to harvest Shiraz had significantly higher ^o^BRIX compared to Cabernet Sauvignon (Figure 1), which was compensated by harvesting Cabernet Sauvignon two weeks later than Shiraz.

### GC/MS based metabolic changes across berry development

**Figure 2:**
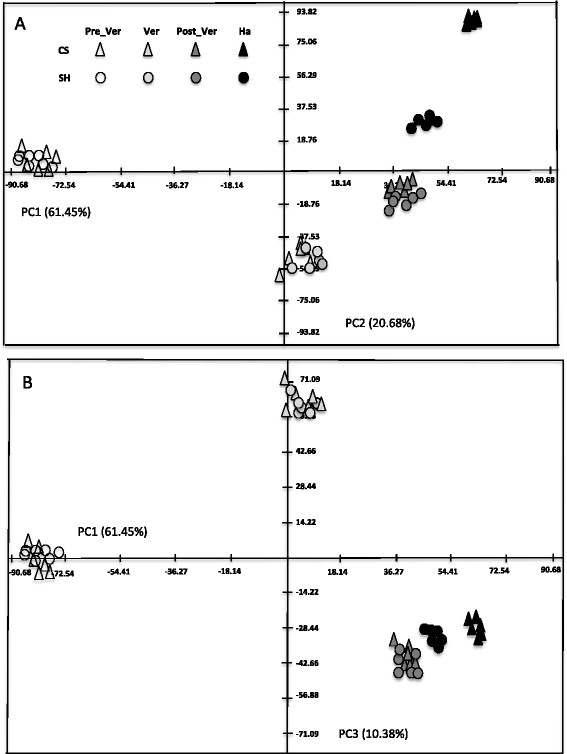
**Metabolic shift of primary metabolism during berry development in Cabernet Sauvignon and Shiraz.** PCA plot of PC1 versus PC2 **(A)** and PC1 versus PC3 **(B)** of metabolite profiles of Cabernet Sauvignon and Shiraz at four berry development stages. Percentage of the variance captured by each PC is given close to each respective axis. Each point in **(A)** and **(B)** represents one biological sample.

In order to visualize the general trend of metabolic changes in respect to the cultivar and developmental stage, all identified 65 central metabolites were subjected to Principal Component Analysis (PCA). Accordingly, the two cultivars exhibited very similar trends of PCA projection (Figure 2) indicating a similar pattern of change in central metabolism. The first three principal components accounted for 92.5% of the total variance and revealed most of the developmental and cultivar variation. The first principal component (PC1) contributed 61.45% of the variance and clearly discriminated developmental stages. Galactonate, pyruvate and leucine had high eigenvalues on PC1, indicating their strong contribution to sample distribution along this component (Additional file 2: Table S2). PC2 described 20.68% of the variance and discriminated the developmental stages veraison, mid-ripening and harvest as well as the two cultivars at harvest. The separation was mostly due to changes in gulonate, dehydroascorbate, butanoate, aspartate, raffinose, fucose, rhamnose, glycine and glycerate (Additional file 2: Table S2). PC3 contributed 10.38% of the total variance, discriminating veraison from post-veraison stages and mainly described by caffeate, maleate, shikimate, fumarate, proline and arginine (Additional file 2: Table S2).

**Figure 3:**
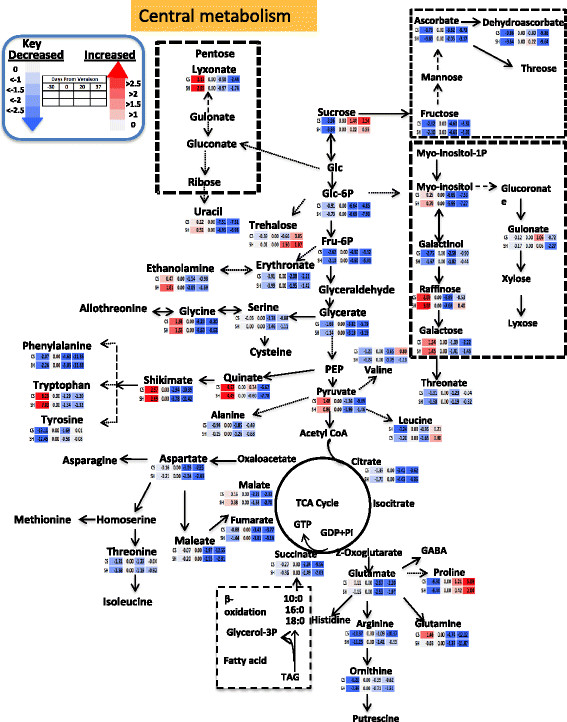
**Schematic representation of primary metabolite pathways with log2 transformed fold change normalized to the veraison stage of each cultivar.** Different colors represent the increase (red) or decrease (blue) of the metabolites fold change as indicated in the color index. n = 6. Each row (upper row CS and lower row Shiraz) metabolite change with reference to veraison stage, where as each column represents different time points (the diagram was generated by using pathway tool program [84]).

Changes in the central metabolites during the four developmental phases were normalized to the veraison stage as a reference point to ease comparison between the two cultivars. Thus the data set was normalized to the values at veraison, log 2 transformed, and the relative changes at pre-veraison, post-veraison and harvest stages in relation to veraison were hence expressed as log2 [fold change]. Nevertheless, it should be noted that direct comparisons between developmental stages of the two cultivars is not possible, rather the cultivars will be compared by investigating the patterns of change of metabolites during berry development. Overall, central metabolites exhibited a similar pattern of change in the two cultivars (Figure 3). The earlier stage of berry development was characterized by high relative abundance of central metabolites compared to later stages with a few exceptions. As expected, sucrose abundance followed an inverse trend accumulating progressively towards harvest. Fructose and phosphate conjugates of fructose and glucose increased toward veraison, later declining towards harvest. Other metabolites including myo-inositol and galactose also peaked at pre-veraison and declined thereafter. Raffinose exhibited the highest abundance at pre-veraison, followed by a steep reduction at mid-ripening and accumulation at harvest. With similar trend of sucrose accumulation in the two cultivars, trehalose progressively accumulated in both cultivars with higher magnitude in Shiraz. Glycolytic intermediates glycerate and pyruvate gradually declined during development in both cultivars but with significantly higher changes between developmental stages in CS compared to Shiraz (Figure 3, Additional file 2: Table S3).

Most amino acids showed progressive reduction towards maturation in both cultivars. However, pyruvate derived amino acids, alanine and valine, had a constant abundance during berry development for both cultivars (Figure 3). Coupled to a pronounced decrease in glycerate, amino acids, serine and glycine, that are derived from glycerate progressively decreased in relative abundance in both cultivars towards harvest but the reduction was higher in Shiraz compared to CS. The 2-oxoglutarate derived amino acids glutamate, arginine, ornithine and glutamine, and the oxaloacetate derived amino acids aspartate and threonine had a progressive decrease in relative abundance in both cultivars. Among shikimate-derived amino acids, phenylalanine showed maximum accumulation at veraison followed by a progressive reduction in relative abundance. The extent of reduction at harvest compared to its level at veraison was somewhat higher in Shiraz compared to CS. The other shikimate-derived amino acids, tyrosine and tryptophan, remained at relatively steady levels following veraison. In contrast to most amino acids, proline relative abundance increased towards maturity in both cultivars (Figure 3).

TCA cycle intermediates, citrate, succinate, fumarate and malate showed similar patterns of change in relative abundance declining progressively towards maturity. However, the extent of decrease was variety dependent. For example, the major organic acid, malate decreased by 6-fold in Shiraz compared to 4-fold reduction in CS. Reduction in citrate level was also higher in Shiraz (~9-fold) compared to CS (7-fold). The lesser abundant organic acids, succinate and maleate [85], decreased toward maturity by 18-fold and 24-fold in CS compared to 4-fold and 6-fold respectively, in Shiraz (Figure 3).

Quinate and shikimate links the primary metabolism to the phenylpropanoid biosynthesis pathways. These metabolites exhibited high reduction across berry development. Quinate and shikimate decreased on average by 14- and 20-fold respectively, at harvest in both cultivars. Cinnamate, the immediate precursor of the phenylpropanoid pathway, showed higher accumulation at pre-veraison followed by a progressive reduction towards maturity in both cultivars.

### LC-MS based metabolic changes across berry development

**Figure 4:**
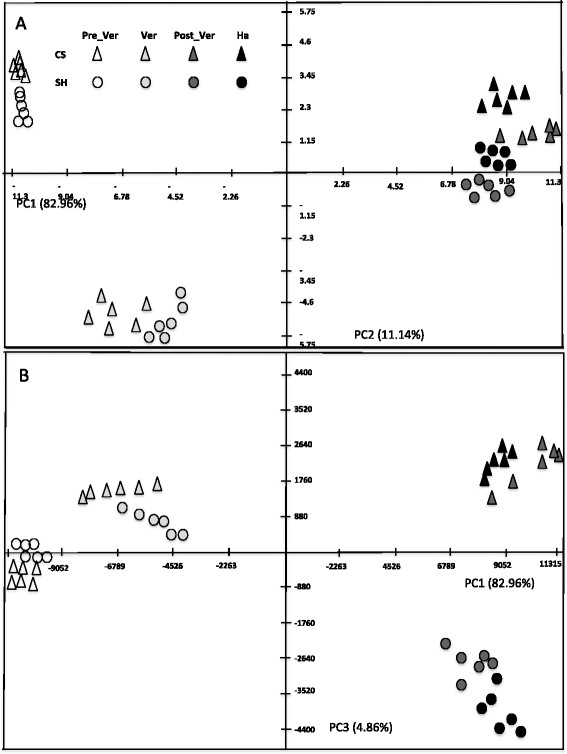
**Metabolic shift of specialized metabolism during berry development in Cabernet Sauvignon and Shiraz.** PCA plot of PC1 versus PC2 **(A)** and PC1 versus PC3 **(B)** of metabolite profiles of Cabernet Sauvignon and Shiraz at four berry development stages. Percentage of the variance captured by each PC is given close to each respective axis. Each point in **(A)** and **(B)** represents one biological sample.

LC-MS based metabolic profiling revealed major changes in the relative abundance of berry-skin specific metabolites during development. Principal Component Analysis (PCA) of the dataset revealed a directional projection, on the first principle component (PC1), of metabolic changes governed by the developmental process in both cultivars (Figure 4); namely PC1 explained the largest variance (82.96%, Figure 4A). PC2 explained 11.14% of the variance corresponding to metabolite changes occurring in the berry-skin between pre-veraison to veraison in both cultivars (Figure 4A). When extracted the eigenvalues of the metabolite relative impact on the distribution of the samples on the PC plot, malvidin 3-*O*-(6″-acetyl-glucoside), malvidin-3-glucoside and malvidin 3-*O*-(6″-*p*-coumaroyl-glucoside) appeared to be the largest contributors to the separation along PC1 (Additional file 2: Table S2). The same metabolites together with tartrate, contributed to data distribution along PC2. Notably, cultivar variability was shown along PC3 but it explained only 4.86% of the variability within the dataset (Figure 4B). Nevertheless we could identify metabolic changes specific to the two cultivars. Respectively, peonidin 3-*O*-(6″-*p*-coumaroyl-glucoside), petunidin 3-*O*-(6″-*p*-coumaroyl-glucoside), malvidin 3-*O*-(6″-*p*-coumaroyl-glucoside) and malvidin 3-*O*-(6″-acetyl-glucoside) contributed to the distribution of the two cultivars on PC3 (Additional file 2: Table S2). When investigating the metabolic data for the cultivar differences indicated by the PCA, the coumaroyl form of anthocyanin was shown to specifically and significantly accumulate in Shiraz at veraison, post-veraison and harvest, while high levels of malvidin 3-*O*-(6″-acetyl-glucoside) relative to Shiraz characterized the berry-skin of CS during these stages. PCA indicated differences in secondary metabolism between the two cultivars, particularly at veraison and ripening stages (Figure 4B). Importantly, the PCA of both primary (Figure 2) and secondary (Figure 4) metabolite profiles grouped together the six biological replicates of each cultivar at the different developmental stages, demonstrating the high level of reproducibility between the replicates compared with the developmental factor.

**Figure 5:**
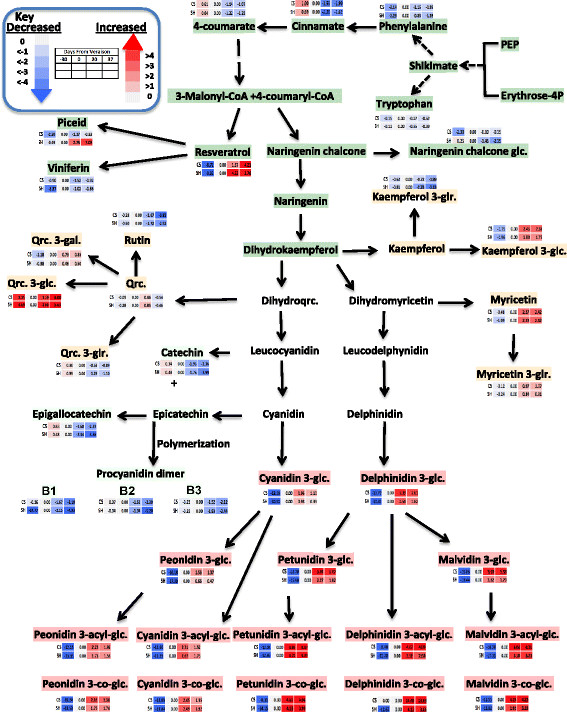
**Schematic representation of specialized metabolite pathways with Log2 transformed fold change normalized to the veraison stage of each cultivar.** Color, row and column keys are the same as Figure 3 (the diagram was generated by using pathway tool program [84]).

The metabolic patterns of change showed that coumarate, the immediate precursor of phenylpropanoid, had greatest accumulation at pre-veraison stage declining afterwards in both cultivars; it is likely incorporated into downstream metabolism (Figure 5). Glycosylated naringenin, naringenin chalcone glucoside, decreased progressively in Shiraz while displaying insignificant changes in CS from veraison to harvest (Figure 5). Hydroxycinamic acid metabolites such as *p*-coumarate, *p*-coumaroyl tartarate, coumaric acid hexoside acid and ferulate reached peak concentration at pre-veraison stage and declined progressively towards maturity (Additional file 1: Figure S2). This is consistent with the previous report by Adams [41]; these metabolites might serve as precursors for the synthesis of volatile and other polyphenolic metabolites.

Stilbenes are a metabolite class that branches from the phenylpropanoid pathway. Stilbenes accumulate in the berry in response to biotic [86]-[91] and abiotic stresses [92]-[94]. The two cultivars have similar patterns of accumulation of the stilbene, resveratrol, which exhibited a progressive accumulation in the berry skin towards maturity. In contrast, the stilbene, viniferin, did not change in abundance during development in both cultivars, while the pattern of change in the glucoside form of resveratrol, piceid, differed between cultivars; it gradually accumulated in Shiraz while keeping at comparable levels in CS following veraison (Figure 5).

Flavonols, the immediate competitors of the precursors for the anthocyanin pathway, showed mild induction following veraison stage with the exception of quercetin glucoside, which accumulated at pre-veraison and post veraison stages with a transient reduction in relative abundance at veraison. Unlike the other flavonols, kaempferol glucuronide and rutin decreased in relative abundance progressively towards maturity (Figure 5). All the flavanol classes (catechin, epicatechin and procyanidin dimers) decreased progressively in abundance towards maturity in both cultivars but the extent of reduction was more intense in Shiraz compared to CS (Figure 5) likely due to competition with the massive anthocyanin accumulation.

Anthocyanins, as expected, displayed progressive accumulation across development in both cultivars. Dihydrokaempferol represents the branching point between different anthocyanin downstream pathways (Figure 5, [95]). With flavonoid 3′-hydroxylase (F3′H), dihydrokaempferol is converted to dihydroquercetin and supports the biosynthesis of glucoside, acetyl and coumaroyl forms of cyanidin and peonidin (cyanidin-type anthocyanins), while with flavonoid 3′,5′ hydroxylase (F3′5′H) it can be converted to dihydromyricetin, that gives rise to the glucoside, acetyl, coumaroyl and caffeoyl forms of delphinidin, petunidin and malvidin (delphinidin-type anthocyanins). Cyanidin and peonidin glucoside biosynthesis had a mild but significant induction in ripening stages after veraison. In both cultivars the results indicated (Figure 5) that all anthocyanins derived from the dihydromyricetin pathway were highly induced compared with the parallel pathway derived from dihydroquercetin (Figure 5). The two cultivars exhibited similar pattern of anthocyanin accumulation with the exception of malvidin 3-*O*-glucoside, which progressively accumulated in CS, and petunidin, which significantly accumulated towards maturity in Shiraz (Additional file 2: Table S3). The two initial glucoside forms, cyanidin glucoside and delphinidin glucoside reached maximum level at mid-ripening and reduced at harvest (Figure 5). Comparing the coumaroyl forms of anthocyanins, Shiraz exhibited remarkably higher levels from veraison onwards (Additional file 1: Figure S3).

### Metabolite correlation and network analysis

To gain an understanding of how metabolites coordinately changed during the season and to identify cultivar-specific metabolite relations, a pairwise correlation analysis was performed among all primary and specialized metabolites as described previously in Hochberg et al. [69]. Metabolites in the same biosynthetic pathway mostly displayed a positive correlation (Additional file 2: Table S4A & S4B). The highest correlations were observed between primary and specialized metabolite classes. Anthocyanins and flavonols exhibited mostly similar trends of correlation, reflected in positive *r* values between the two classes and in negative *r* values with organic acids and most of other identified primary metabolites. Particularly, anthocyanins displayed strong negative relationship with flavanols, flavanones and hydroxycinamic acids and other organic acids. The Shiraz data show a greater number of significant correlations between metabolites than CS (Additional file 2: Table S4A & S4B, Additional file 1: Figure S4A).

Metabolic networks generated from matrices of significant correlations (Q < 0.05 and 0.7 < r < −0.7) built for Shiraz and CS indicated an overall higher number of edges (1957 vs 1771, respectively), average nodal degree (39.5 vs 34.7, respectively) (Additional file 2: Table S4C) and frequency of the nodes with large degree in Shiraz network compared to CS (Additional file 1: Figure S4B). Taking together, the lower diameter of the Shiraz network (Additional file 2: Table S4C) suggests a tighter coordinated behavior of metabolism during the development of Shiraz compared to CS. This suggestion is also supported by higher transitivity score of the Shiraz network (Additional file 2: Table S4C), i.e. the probability of a network to form clusters with stronger interconnections.

### Transcriptomic changes during berry development

Sampling berries with similar developmental stages can be difficult due to variability even between clusters on the same vine and among berries within clusters. Precautions were taken to collect representative and average (in size and appearance) berries from the middle of the cluster to reduce such developmental variations. Veraison is a transitional phase from green stage to ripening when massive metabolite changes towards ripening begin [15],[46],[65],[96] and this transition brings a fundamental transcriptomic reprograming of the entire vine [56] that determines the final metabolite composition of the berry. Taking into consideration the results of the whole-berry weight and biochemical parameters and to minimize the developmental element in the variation of gene expression between the two cultivars, samples for RNA-seq data analysis were harvested at veraison and 20 days after veraison (DAV), i.e. maximal anthocyanin content (Figure 1). At both developmentally and biochemically defined stages, the two cultivars displayed similar ^o^BRIX levels, which made the comparative investigation of dynamics of transcriptional and metabolite changes possible [57]. Following identification of significantly changed transcripts, to facilitate the comparison and visualization of transcriptomic changes in both cultivars, the data were normalized to the CS veraison stage transcript level to visualize the change between stages and between cultivars.

**Figure 6:**
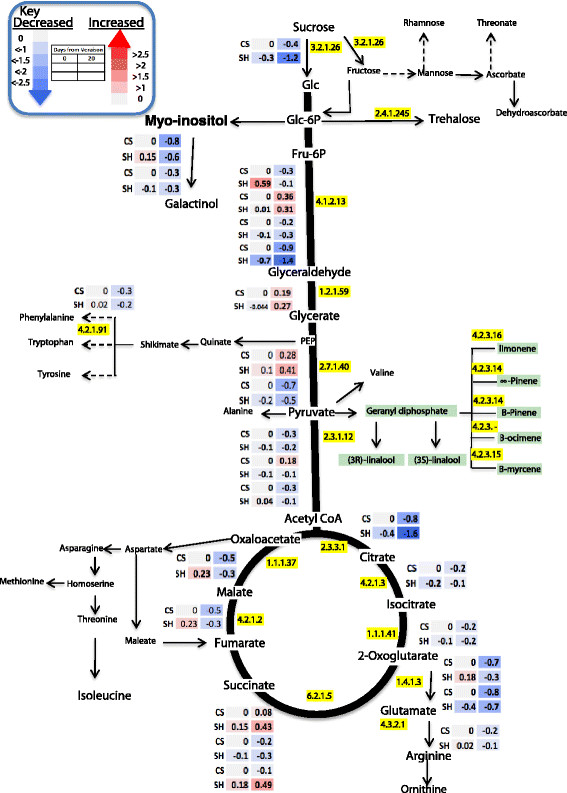
**Berry skin transcripts involved in the primary metabolism and changing significantly from veraison to post veraison stage in Shiraz and CS.** The data were normalized to the respective transcript abundance in CS veraison stage. Heatmaps represent changes in transcript expression levels (log_2_ transformed) as indicated by the color legend and by the values within each box. EC numbers for the mentioned enzymes are: 3.2.1.26 (Fructofuranosidase), 2.4.1.245 (Trehalose synthase), 4.1.2.13 (Fructose-bisphosphate aldolase), 2.7.1.40 (Pyruvate kinase), 1.2.1.59 (Glyceraldehyde-3-phosphate dehydrogenase), 2.7.1.40 (Pyruvate kinase), 2.3.1.12 (Acyl-CoA:dolichol acyltransferase), 4.2.3.16 (Limonene synthase), 4.2.3.14 (Pinene synthase), 4.2.3.15 (Myrcene synthase), 2.3.3.1 (Citrate (Si)-synthase), 4.2.1.3 (Aconitase), 1.1.1.41 (Isocitrate dehydrogenase), 1.4.1.3 (Glutamate dehydrogenase), 4.3.2.1 (Argininosuccinate lyase), 4.2.1.5 (Arabinonate dehydratase), 6.2.1.5 (Succinyl-CoA synthetase), 4.2.1.2 (Fumarate hydratase), 1.1.1.37 (Malate dehydrogenase) 4.2.1.91 (Arogenate dehydratase) (the diagram was generated by using pathway tool program [84]).

Additional file 2: Table S5 displays a list of transcripts that significantly changed as the berry grew from veraison to mid-ripening stage. Among transcripts coding for carbohydrate metabolism most of the sucrose synthase genes had modest down-regulation while a transcript putatively encoding for conversion of sucrose to glucose (VIT_07s0005g00690) decreased significantly in both cultivars from veraison to mid-ripening stage (Figure 6). A gene putatively coding a beta-fructofuranosidase (VIT_06s0061g01440) that facilitates sucrose degradation decreased significantly in CS from veraison to 20 DAV. Instead, Shiraz displayed a different trend in regulating sucrose degradation. Among sucrose degrading genes, VIT_14s0060g00770, was significantly upregulated while the other gene, VIT_06s0061g01440, was significantly down regulated, indicating differential expression during the early and late ripening stages in sugar metabolism (Additional file 2: Table S5). A gene coding for trehalose phosphatase (VIT_01s0026g00220) was highly induced in Shiraz (3.3-fold change) compared to CS (1.7-fold change for VIT_02s0012g01620). In contrast, CS showed a down regulation of a different gene in trehalose metabolism coding for trehalose synthase (VIT_06s0009g01580).

Transcripts putatively encoding for TCA cycle enzymes such as citrate synthase (VIT_13s0156g00110) aconitase (VIT_05s0049g01980), isocitrate dehydrogenase (VIT_14s0066g00950), succinyl-CoA synthetase (VIT_10s0042g00950 and VIT_01s0127g00260) and fumarate hydratase (VIT_07s0005g00820) were downregulated following veraison (Figure 6), consistent with the metabolite profiles of these TCA intermediates.

**Figure 7:**
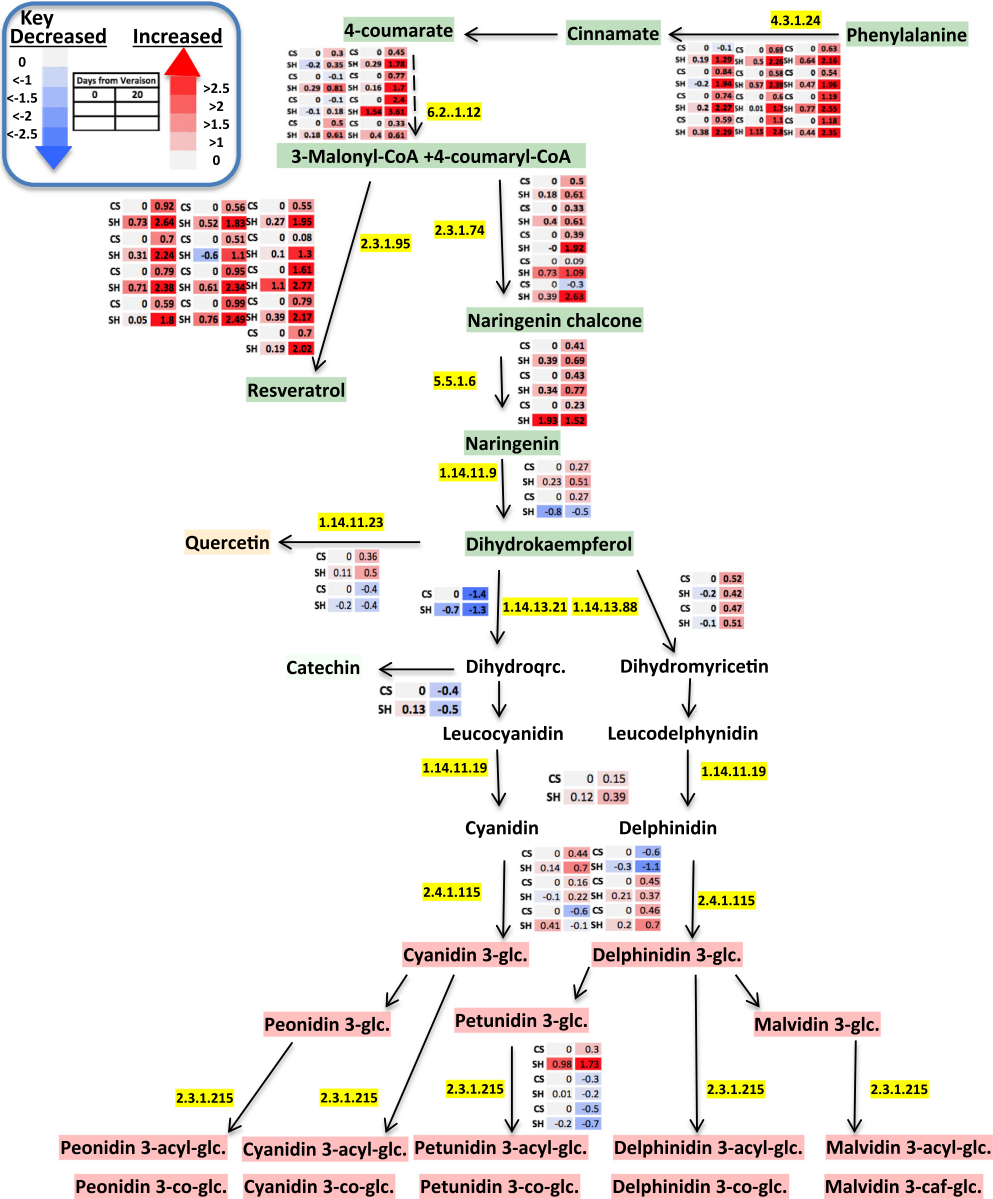
**Changes in transcript level of flavonoid genes in the berry skin of Shiraz and CS from veraison to post veraison stage.** The data were normalized to the respective transcript abundance in CS veraison stage. Heatmaps represent changes in transcript expression levels (log_2_ transformed) as indicated by the color legend and by the values within each box. EC numbers for the mentioned enzymes are: 4.3.1.24 (phenylalanine ammonialyase), 6.2.1.12 (4-coumarate---CoA ligase), 2.3.1.74 (Naringenin-chalcone synthase), 2.3.1.95 (Stilbene synthase), 5.5.1.6 (chalcone isomerase), 1.14.11.9 (Naringenin 3-hydroxylase), 1.14.11.23 (Flavonol synthase), 1.14.13.21 (Flavanoid 3′-hydroxylase), 1.14.13.88 (Flavanoid 3′,5′-hydroxylase), 1.14.11.19 (Anthocyanidin synthase), 2.4.1.115 (Anthocyanidin 3-O-glucosyltransferase) and 2.3.1.215 (Anthocyanidin 3-O-glucoside 6″-O-acyltransferase) (the diagram was generated by using pathway tool program [84]).

The phenylpropanoid pathway provides precursors for stilbenes and flavonoids. However, no significant variation was observed in the expression levels of cinnamate-4-hydroxylase (C4H) genes, the enzyme responsible for catalyzing the 4-hydroxylation of *trans-*cinnamate. Nevertheless, five genes putatively involved in phenylpropanoid biosynthesis, 4-coumaroyl-CoA ligase (4CL), chalcone synthase (CHS), chalcone isomerase (CHI) and flavonoid 3′, 5′-hydroxylase (F3′5′H), were co-regulated and differentially expressed from veraison to 20 DAV in both cultivars (Figure 7). Moreover, the expression of transcripts coding for phenylalanine ammonia lyase (PAL), was significantly upregulated in Shiraz to a much greater extent than in CS, supporting the intense utilization of the precursor metabolite for the downstream biosynthesis during development in that cultivar. Genes involved in the subsequent biosynthesis step that lead to 4-coumaryl-CoA were also induced in both cultivars, but to a larger extent in Shiraz, supporting the substantial accumulation of the coumaroyl form of anthocyanins in Shiraz.

Differentially accumulated transcripts involved in the phenylpropanoid and flavonoid pathway are depicted in Figure 7. Interestingly genes involved in the hydroxylation of dihydrokaempferol via putative flavonoid 3′,5′ hydroxylase (F3′5′H) (VIT_06s0009g02970 and VIT_06s0009g02840) that lead to anthocyanin precursor, delphinidin, significantly increased as the berry developed from veraison to post-veraison stage in both cultivars. In contrast, the parallel biosynthetic pathway that utilizes the same precursor, dihydrokaempferol to another anthocyanin precursor (cyanidin) via the enzyme flavonoid 3′ hydroxylase (F3′H) did not show significant increased expression in Shiraz while being downregulated in CS from veraison to mid-ripening stage (Additional file 2: Table S5). Previous studies showed that high expression of F3′H and F3′5′H correlated with the composition of flavonols, flavanols and some anthocyanins [97],[98]. Hence, F3′H as an early acting flavonoid gene can provide precursor for flavanol and cyanidin type of anthocyanin whereas F3′5′H might divert the flux to delphinidin derivatives at later stages of berry development. Thus, the significantly higher expression of F3′5′H transcripts at postharvest is presumably involved in polyphenol maturation. Our analysis of metabolites and transcripts suggest a consistent association between metabolites and related gene transcripts in the polyphenol biosynthesis (Figures 5 and 7).

The cuticular membrane of grape berry, which is composed of a wax layer that protects the fruit from desiccation and injuries. Three putative wax synthase-coding transcripts (VIT_15s0046g00590, VIT_15s0046g00520 and VIT_03s0063g00050) decreased significantly in abundance from veraison, while three other wax synthase-coding transcripts (VIT_15s0046g00470, VIT_19s0090g01340 and VIT_19s0090g01350) were induced significantly as the berry developed from veraison to the mid-ripening stage in CS; this was not so in Shiraz berry skins, where a down regulation of two of the wax-synthesis related genes (VIT_15s0046g00590 and VIT_15s0046g00520) was detected and only one was significantly upregulated (VIT_15s0046g00470) (Additional file 2: Table S5). Our results partly contrast with previous microarray transcriptomic studies where maximum wax synthase-coding transcripts were shown at veraison [47],[99]. The two cultivars exhibited differential expression in two different transcripts coding for universal stress protein (USP). Shiraz exhibited greater changes (3.2- and 2.4-fold changes for VIT_06s0004g05670 and VIT_08s0007g01360, respectively) compared to changes in CS (2.4- and 1.7-fold changes for VIT_07s0005g01290 and VIT_08s0007g01360, respectively) (Additional file 2: Table S5). These data are in line with previous results where we showed a more pronounced drought related response in Shiraz to progressive water deficit compared to CS [69].

Expectedly, many phytohormones related genes involved in the berry ripening process showed changes in transcription level (Additional file 2: Table S6) in both cultivars as the berry developed from veraison to post-veraison stage. For example, a total of 19 genes related to ABA metabolism showed differential expression in development and cultivar specificity, i.e. an upregulation of ABA related genes was shown in Shiraz, whereas a repression of the same process was evident in CS (Additional file 2: Table S6). The expression of genes related with ethylene biosynthesis also highly changed in expression during development while showing similar trends in the two cultivars (Additional file 2: Table S6). Similarly, brassinosteroid biosynthesis related genes were shown to change significantly in the two cultivars but to different extents. Namely, in CS, two genes upregulated and eight genes downregulated while in Shiraz three genes were upregulated while five were downregulated. In relation to jasmonate biosynthesis, the two cultivars upregulated one gene, while showing a greater downregulation of the process in CS (five genes) than Shiraz (two genes, Additional file 2: Table S6).

### Gene enrichment analysis

The results of functional classification on classified genes using Expander enrichment analysis tool are shown in Additional file 1: Figure S5. The enrichment analysis yielded 44 and 37 significantly enriched networks in CS and Shiraz, respectively. The detail list of significantly enriched networks (Bonferroni FDR corrected *p-* value cutoff: 0.05) and member of gene lists with a further partitioning into upregulated and downregulated genes, is given in Additional file 2: Table S7. Among the classified genes, Shiraz exhibited higher enrichment in carbohydrate and specialized metabolic processes, whereas lipid, developmental and DNA/RNA processes were over represented in CS berries (Additional file 1: Figure S5).

## Discussion

Cultivar differences in berry metabolism and its regulation by endogenous and environmental cues during development give rise to the distinct varieties in wine flavor [21],[22],[100]-[102]. Light, temperature and water demand are significant factors affecting plant growth and development [103]-[105] . In this respect, Cabernet Sauvignon and Shiraz were recently shown to harbor different hydraulic behavior and water use [106], confirmed in the field in the present study measuring the volumetric soil water content (Additional file 1: Figure S1), and responded differently to terminal drought in respect to leaf metabolism and vegetative physiology [69]. Previously, separate investigations on berry skin microarray analysis of Cabernet Sauvignon [15] and RNAseq of Shiraz [57] reported transcriptional profile and dynamics of gene expression during the course of berry development. Here a comparative parallel analysis of metabolites and RNA transcripts by RNAseq of the two cultivars enabled a comprehensive description of the commonalities and differences in the regulation of the metabolic processes during berry development.

During the early stages of berry development the berry skin is entirely green and photosynthetically active [107],[108], contributing locally to the biosynthesis of sugars and other organic acids [65],[109] that will later be used as substrate in the specialized metabolism. Following veraison, however, a progressive decline in the sugar content, as here shown, is known to occur in support of a continuous use of carbohydrate resources produced via photosynthesis as recently reported by Dai et al. [65]. Most of the identified organic acids showed progressive decrease in both cultivars from veraison to berry ripening. The metabolite profiles were supported by the transcript profiles indicating a down regulation of the TCA cycle in both CS and Shiraz as the berry developed from veraison to 20 DAV. Our results are in accordance with previous findings [57] indicating that the progressive decline of TCA cycle activity likely matches the increased demand of supply for precursors in the synthesis of carbohydrates, amino acids and subsequent flavonoids [65]. Nevertheless a larger carbon demand may be channeled into the specialized metabolism of Shiraz and specifically in the enhanced production of anthocyanins, particularly coumaroyl forms (discussed below). For example greater sucrose accumulation was exhibited in CS than in Shiraz from veraison to maturation; lower accumulation of quinate and shikimate, precursors of the polyphenol metabolism, were measured in Shiraz during later stages of maturation, suggesting an increased integration into downstream processes (anthocyanins production). Correlation analysis further supported the evidence of highly coordinated metabolic changes between central and specialized metabolism. The occurrence of a greater number of significant correlations in the Shiraz metabolite profiling, the stronger properties of the metabolic network of Shiraz compared with CS and the relatively higher expression of flavonoid genes suggest that Shiraz metabolism has a relatively tighter coordination of the metabolic pathways investigated.

The progressive development to maturation was characterized by the increased expression of a high number of transcripts putatively encoding for phenylalanine ammonia lyase (PAL), stilbene synthase, chalcone synthase and chalcone isomerase; these were upregulated in both cultivars, but more significantly in Shiraz. Concomitantly, at the metabolite level, shikimate content was substantially reduced. An intermediate metabolite that links primary metabolism to the polyphenol biosynthesis [16],[110], shikimate is the precursor of aromatic amino acid, phenylalanine, which was decreased in relative abundance towards maturity in coordination with higher content of downstream polyphenols. Changes in metabolite (Phe) and related transcripts in Shiraz were greater than CS, indicating a coordinated shift of central metabolism fueling the biosynthesis of specialized compounds in both cultivars, but to a greater extent in Shiraz. The continuous supply of nutrients during ripening via phloem from matured leaves fuels berry metabolic processes [111]. Nevertheless, it is tempting to hypothesize that the commencement of specialized metabolism in the skin at veraison is supported by the biosynthesis and accumulation of primary substrates, e.g. sugars and organic acids including shikimate, via localized production of photoassimilates during the pre-veraison stages. The role of photosynthesis in the fleshy fruits was addressed recently in developing tomatoes [112]. The results showed that at least under the conditions used fruit photosynthesis was not necessary for fruit energy metabolism or development, but it was necessary for properly timed seed development. The role of photosynthesis in organs shifting from autotrophic to hetrotrophic tissue was investigated in green seeds too. Here the results showed that photosynthesis contributed to maintain energy and oxygen levels over a metabolic-promotive threshold in a tissue that subsequently would become dense and hypoxic [113]. These contradicting results prompt for more work to elucidate the role of localized photosynthesis to tissue development. Berry maturation was also accompanied by enhanced stress related metabolism such as trehalose, stilbene and ABA suggesting for a more susceptible Shiraz to environmental cues, likely related with the conditions in the field, reflected in high radiation and temperature. In support of this hypothesis in a recent study under progressive drought conditions Shiraz responded with greater alterations in the central metabolism of the leaf and by the accumulation of stress related compounds [69].

### The two cultivars exhibited similar patterns in transcripts and metabolites of the flavonoid pathway, but differed in their magnitude of change

Anthocyanins make up a significant portion of berry skin flavonoids [114] and five classes (cyanidin, peonidin, delphinidin, petunidin and malvidin) are commonly found in *Vitis vinifera* grape with their glucosides, acetyl-glucosides, coumaroyl-glucosides, peonidin and malvidin caffeoyl-glucosides [95]. Dihydrokaempferol is a common substrate for F3′H and F3′5′H to produce their corresponding cyanidin-type and delphinidin-type anthocyanins [21]. The two enzymes, F3′H and F3′5′H, belong to the cytochrome p450 protein family [115] and compete a common precursor for the synthesis of red and blue anthocyanins, respectively [116]. Previous phylogenetic analysis indicated that F3′5′H was recruited from F3′H before the divergence of angiosperms and gymnosperms [117]. In the current study transcripts coding for F3′5′H increased significantly in relative abundance from veraison to post veraison stages in both cultivars. The increase in F3′5′H transcript abundance was coupled to a decrease of F3′H transcript abundance and with higher accumulation of delphinidin-type anthocyanins in the downstream steps of this pathway. Presumably F3′5′H plays a key role in driving the phenylpropanoid substrate flux towards delphinidin-type anthocyanin pathways. Mattivi et al. [21] also suggested that higher F3′5′OH activity would direct anthocyanin modification towards delphinidin-type anthocyanin forms. Liu et al. [118] demonstrated the greater increase in transcript abundance of F3′5′H genes under UV treatment compared with the changes in transcript abundance of F3′H genes in Antarctic moss. Perhaps, F3′5′H genes are highly associated with strategies to cope with increased stress during berry ripening.

In the present study the coumaroyl form of anthocyanins were accumulated in Shiraz to a significantly higher extent compared to CS (Additional file 2: Table S8) towards the later stage of berry development and this coincided with the increase in the level of phenylpropanoid-related gene transcripts. The acylation step of anthocyanin biosynthesis is mediated by anthocyanin acyltransferase which links aromatic constituents to the C6′ position of the glucosyl group [119]. This step of biosynthesis promotes color stability and intensity of anthocyanins [120]-[122]. Our results are in agreement with substantial cultivar variability in coumaroyl forms of anthocyanins as previously reported by Mazza et al. [123] and Boss et al. [124].

### Shiraz showed enhanced stilbene metabolism

A significant cultivar specificity was observed in the stilbene metabolism and regulation. Specifically, Shiraz displayed a significant upregulation of many STS genes during ripening, which was not observed in CS. The transcript profile was in accordance with the content of resveratrol and piceid, which accumulated in Shiraz more than in CS. These findings indicate a differential regulation of stilbene metabolism between the two cultivars and confirm the high correlation of STS transcript level and stilbene compounds abundance in grape berry skin [125],[126]. Having said that, the branching between stilbene and flavonoid metabolism may imply a competition for the same precursors. However the concomitant accumulation of anthocyanins in Shiraz suggests that the entire polyphenol pathway is upregulated in this cultivar compared with CS, possibly as a result to local climate conditions (expressed by the stress related genes and metabolites) and at the expense of primary C substrates (e.g. sucrose).

### Shiraz exhibited enhanced transcription of phytohormone related genes

The role of phytohormones in relation to berry ripening with their coordinated interaction with other signal molecules has been reported previously [127]. Exogenous ABA application induces the ripening process in climacteric fruits such as tomato [128],[129] and banana [130]. Endogenous ABA was associated with sugar metabolism in water stressed grapevine [131]. Previous work demonstrated that the level of ABA in the berry increase towards ripening [11],[132],[133] and it is likely involved in enhanced phenolic compound biosynthesis [134]-[137]. Our data indicates upregulation of ABA related genes in Shiraz and indeed associated with varietal specific enhanced anthocyanin accumulation and specifically coumaroyl anthocyanin forms. Cramer et al. [138] demonstrated that among the genes related to hormone metabolism, ABA related transcripts changed the most in response to water and salinity stresses. Given the recently established differences in stress response between Shiraz and CS [69],[106], a possible explanation for the enhanced ABA-related genes and other stress related processes, could be related to magnitude of stress experienced by that cultivar under the condition of growth in the experimental field, e.g. high evapotranspiration and radiation and low humidity.

## Conclusion

The present comparative study showed a tight coordination between polyphenol metabolism and respective transcripts in the developing berries of the two cultivars. The inverse regulation of the two committing steps of phenylpropanoid metabolism, F3′H and F3′5′H, sharing a common substrate, was reflected by the metabolite profiles of the two parallel downstream anthocyanin pathways. Correlation analysis showed a “source sink”- like relation between central and specialized metabolism, also supported by the transcript analysis. At the variety level, Shiraz was characterized by a more synchronized developmental metabolism as well as by a greater upregulation of the entire polyphenol pathway, stress related processes. Taken together these data indicate that the varietal susceptibility to the environment may be influenced by the vines hydraulic behavior and physiology, and may determine important metabolic traits at fruit harvest.

### Availability of supporting data

Supporting data for list of metabolite standards, principle component analyses, metabolite correlation analysis, list of significantly changed transcripts, list of phytohormones related genes, and enrichment analysis, metabolite pairwise comparison of samples and statistical analysis of coumaroyl anthocyanin forms are available in Additional file 2: Tables S1–S8. The change in volumetric water content of the soil, relative abundance of hydroxycinamate, relative abundance of coumaroyl anthocyanin forms, summary of correlation analysis and functional category distribution are presented in Additional file 1: Figures S1–S5.

## Competing interests

The authors declare that they have no competing interests.

## Authors’ contributions

AD conceived and conducted the experiment, analyzed LCMS data and wrote the body of the paper with AF; UH helped running the experiment, processed the samples for metabolite profiling and analyzed GCMS data. NS provided technical support in UPLC machine sample run, LV participated in the sequence assembly and the subsequent RNAseq computational analysis, GB performed sample preparation for sequencing and the sequencing itself, RG helped in RNAseq analysis, AB performed network analysis, IP and VC participated in RNAseq and enrichment analysis, FM helped in metabolite verification using standards, MD, MP and GRC helped in RNAseq data analysis, interpretation and edit the manuscript, AF conceived and coordinated the project together with SR. All authors reviewed, edited and approved the final version of the manuscript.

## Additional files

## Supplementary Material

Additional file 1: Figure S1. Change in volumetric water content of the soil in CS and Shiraz cultivars during the irrigation cycles. **Figure S2.** Relative abundance (log10 scale) of coumaroyl tartarate coumaric acid hexoside and ferulate at four developmental stages of the two cultivars. Values represent mean ± SE (n = 6). **Figure S3.** Coumaroyl form of berry skin anthocyanin at four developmental stages of the two cultivars. Values represent mean ± SE (n = 6). **Figure S4A.** Summary of metabolite correlation in CS and Shiraz with ‘+’ and ‘–’ indicating significant (p < 0.05) positive (r > 0.7) and negative (R < −0.7) correlations, respectively, **Figure S4B.** Nodal degree distribution of CS (left) and Shiraz (right) networks. **Figure S5.** Functional category distribution of differentially expressed gene transcripts (classified) of CS and Shiraz. Transcripts were grouped into 12 (CS) and 10 (Shiraz) higher level functional categories. The entire list of categories generated using Expander enrichment analysis tool is presented in Table S5. Click here for file

Additional file 2: Table S1. List of metabolite standards and retention time (min) run in UPLC-qTOF machine. **Table S2.** Statistical data of the PCA (Principle Component Analysis) of GC-MS and LC-MS based metabolites including loadings and% of variance explained. **Table S2. Table S3A.** Kruskal-Wallis and pairwise comparisons of Wilcoxon rank sum test values of CS. Mean values are average of six biological replicates. **Table S3B.** Kruskal-Wallis and pairwise comparisons of Wilcoxon rank sum test values of Shiraz. Mean values are average of six biological replicates. **Table S4A and S4B.** Pairwise Pearson correlation matrix of all annotated metabolite, bolded figures represent significant correlation at p < 0.05 in CS (A) and Shiraz (B) **Table S4C.** Correlation-based networks properties of CS and Shiraz grape cultivars. **Table S5.** List of significantly changed transcripts in Cabernet Sauvignon and Shiraz berry skin as the fruit develop from Veraison to post-veraison stage. **Table S6.** List of differentially expressed phytohormons in CS and Shiraz berry skin as the fruit develop from veraison to post-veraison stage. **Table S7.** Functional categories of significantly changed transcripts in CS (CS) and Shiraz (B) berry skin. **Table S8.** Statistical analysis of coumaroyl anthocyanins forms at four berry developmental stages in the two cultivars. Click here for file
